# A Review of the Impact of Implant Biomaterials on Osteocytes

**DOI:** 10.1177/0022034518778033

**Published:** 2018-06-04

**Authors:** F.A. Shah, P. Thomsen, A. Palmquist

**Affiliations:** 1 Department of Biomaterials, Sahlgrenska Academy, University of Gothenburg, Göteborg, Sweden

**Keywords:** osseointegration, bone, dental implants, bone matrix, biocompatible materials, bone-implant interface

## Abstract

In lamellar bone, a network of highly oriented interconnected osteocytes is organized in concentric layers. Through their cellular processes contained within canaliculi, osteocytes are highly mechanosensitive and locally modulate bone remodeling. We review the recent developments demonstrating the significance of the osteocyte lacuno-canalicular network in bone maintenance around implant biomaterials. Drilling during implant site preparation triggers osteocyte apoptosis, the magnitude of which correlates with drilling speed and heat generation, resulting in extensive remodeling and delayed healing. In peri-implant bone, osteocytes physically communicate with implant surfaces via canaliculi and are responsive to mechanical loading, leading to changes in osteocyte numbers and morphology. Certain implant design features allow peri-implant osteocytes to retain a less aged phenotype, despite highly advanced extracellular matrix maturation. Physicochemical properties of anodically oxidized surfaces stimulate bone formation and remodeling by regulating the expression of RANKL (receptor activator of nuclear factor–κB ligand), RANK, and OPG (osteoprotegerin) from implant-adherent cells. Modulation of certain osteocyte-related molecular signaling mechanisms (e.g., sclerostin blockade) may enhance the biomechanical anchorage of implants. Evaluation of the peri-implant osteocyte lacuno-canalicular network should therefore be a necessary component in future investigations of osseointegration to more completely characterize the biological response to materials for load-bearing applications in dentistry and orthopedics.

## Osteocytes Are Key Determinants of Bone Structure and Function

**Figure 1.**
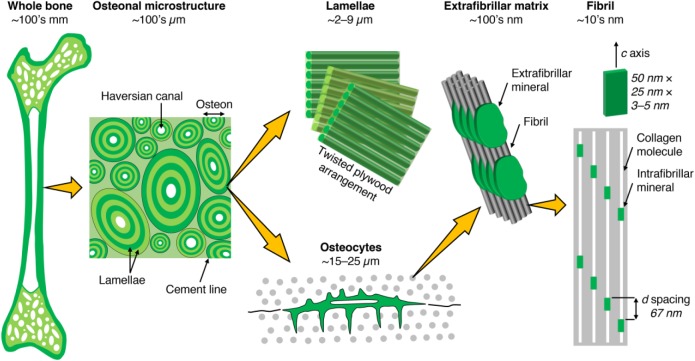
Bone consists of either a porous trabecular framework or a dense cortical structure. In cortical bone (e.g., in the middiaphysis of the femur), the microstructure consists of osteons (170- to 250-µm diameter), which are the units of bone produced during remodeling. Osteons contain a central vascular canal, the Haversian canal (60- to 90-μm diameter), concentrically surrounded by lamellae having a twisted plywood arrangement, where neighboring lamellae have different fibril orientations. Osteocytes reside in lacunae interconnected through canaliculi (100- to 400-nm diameter). Lamellae are composed of collagen fibrils (80- to 100-nm diameter). Fibrils are surrounded by extrafibrillar mineral platelets. Within the fibrils, type I collagen molecules and carbonated apatite crystallites form a nanocomposite structure.

The osteocyte is a central component within the complex hierarchical architecture of bone (Fig. 1). Approximately 42 billion osteocytes reside within the average human skeleton, of which ~9 million are replaced throughout the skeleton every day (Buenzli and Sims 2015). Distributed throughout the mineralized extracellular matrix (ECM), osteocytes play critical roles in bone formation and remodeling, where osteocyte-driven control of bone formation is through the *SOST*/sclerostin mechanism (Robling et al. 2008), while osteocyte-driven control of bone remodeling is through the signaling mechanism involving RANKL (receptor activator of nuclear factor–κB ligand), RANK, and OPG (osteoprotegerin; Nakashima et al. 2011). The RANKL/OPG ratio correlates with histomorphometric indices for physiologic bone remodeling (i.e., eroded surface and osteoid surface; Fazzalari et al. 2001). Sema3A (semaphorin 3A) binding to Nrp1 (neuropilin 1) has been shown to simultaneously suppress osteoclastic bone resorption and promote osteoblastic bone formation through modulation of RANKL and the canonical Wnt/β-catenin signaling pathway, respectively (Hayashi et al. 2012). Furthermore, osteocytes may control perilacunar/canalicular remodeling through transforming growth factor β signaling (Dole et al. 2017).

Alignment of the lacuno-canalicular network closely follows the pattern of bone formation (Kerschnitzki et al. 2011) and correlates directly with mineral particle thickness and orientation (Kerschnitzki et al. 2013). In human osteonal bone, the average length of the canalicular network is ~0.074 ± 0.015 μm/μm^3^ (Repp et al. 2017). Formation of organized lamellar tissue is preceded by the formation of disordered woven bone (Liu et al. 2010). The process is assisted by the presence of a substrate layer on to which osteoblasts can assemble, align, and produce an ordered tissue where collagen fibrils are arranged in parallel over distances beyond the range of a single cell (Kerschnitzki et al. 2013). The secretory territory of rat osteoblasts on the parietal bone is only 154 µm^2^ per osteoblast (Jones 1974). Therefore, spatial organization of the ECM over a length scale corresponding to the size of many matrix-producing cells requires coordinated activity of bone-forming cells, further highlighting that the initial spatial arrangement of these cells is a critical determinant (Kerschnitzki et al. 2013).

In osteonal bone, between concentrically arranged cell layers are highly organized bone lamellae with near-parallel collagen fiber orientation. In older bone, canaliculi appear disrupted due to the remodeling process and deposition of a cement line delineating the interface between older and newer osteons. Canalicular density is greatly reduced in the vicinity of cement lines, confirming the notion that the surrounding old bone serves merely as a substrate for new bone deposition but does not necessarily guide the process through molecular signaling (Kerschnitzki et al. 2013).

Primarily formed woven bone contains 40% to 100% more osteocytes (depending on the skeletal site) than lamellar bone (Hernandez et al. 2004); it is disordered and lacks a predominant mutual alignment between osteocytes; and the canaliculi are directed radially from the lacuna toward the neighboring cells. As a result of fewer canaliculi, connectivity among osteocytes is reduced in comparison with organized lamellar bone. Moreover, collagen fibrils lack a long-range order, as they are arranged concentrically around the osteocytes and are thus perpendicular to the radial canaliculi alignment. This organization of the collagen matrix can be defined as a microlamellar arrangement. The osteocytes in secondarily formed lamellar bone are mainly aligned in layers along bone lamellae, which are connected by canaliculi running perpendicularly through those layers. This difference in organization suggests that a single osteoblast will organize the tissue only within a certain radius of action that is on the same order of magnitude as the spacing between osteocytes in microlamellar bone, ~20 to 30 µm (Sugawara et al. 2005). However, when osteoblasts are supported by a substrate—such as a layer of poorly organized bone, a cement line, or a biomaterial surface—they are able to coordinate their activity, such as to synthesize a layer of parallel-ordered collagen over distances considerably larger than a single cell (Shapiro 2008).

Parameters such as microstructure and ECM composition play an important role in establishing whether the bone formed around implants is healthy and mechanically competent. The bone-implant interface must be understood as a wide zone in which many complex physical and chemical interactions take place between the inanimate implant surface and the surrounding physiologic system. At the interfacial zone between metal implants and bone, in addition to the organic and inorganic building blocks of the ECM, one component that has been frequently overlooked is the osteocyte.

Osteocytes are mechanotransducers and important determinants of bone quality. Through their primary cilia (Nguyen and Jacobs 2013) and cellular processes (Thi et al. 2013), osteocytes are highly mechanosensitive and alter the production of a multitude of signaling molecules triggered with a mechanical stimulus, enabling them to locally modulate osteoblast and osteoclast activity in vitro (You et al. 2008). However, in vivo, osteocyte density declines in association with accumulation of microdamage and with advancing age (Vashishth et al. 2000). Osteocyte survival is a significant determinant of ECM volume (Vashishth et al. 2002), and a strong association exists between decreased osteocyte density and increased porosity (Hunter and Agnew 2016). Moreover, osteocyte lacunar porosity can be used to predict bone matrix stiffness (Yeni et al. 2001).

Osteocytes express a diverse set of genes and proteins. The earliest marker known to be expressed by osteocytes is the membrane-bound protein E11 (or podoplanin), which binds to CD44. Macrophage-capping protein, destrin, E11, and CD44 function to regulate cytoskeletal arrangement and the formation of dendritic processes. FGF23 (fibroblast growth factor 23) regulates serum phosphate levels. FGF23 with PHEX (phosphate-regulating gene with homologies to endopeptidases on the X chromosome) and MEPE (matrix extracellular phosphoglycoprotein) regulate phosphate homeostasis. DMP1 (dentin matrix acidic phosphoprotein 1) plays important roles in regulation of mineralization and osteocyte maturation through regulation of phosphate homeostasis. Encoded by the *SOST* gene, sclerostin expression is confined to mature osteocytes and is a negative regulator of bone formation and, through its regulation of PHEX and MEPE, regulates the differentiation from late osteoblast to preosteocyte (Dallas et al. 2013).

## Osteocytes and Implant Biomaterials

Through the presence of osteocytes adjacent to the implant surface after prolonged healing periods, early investigators demonstrated that peri-implant bone around metals is indeed a living tissue (Serre et al. 1994). The inner wall of the osteocyte lacuna exhibits basophilic staining properties similar to the osteoid-like matrix interposed between mineralized bone and the implant surface (Piattelli et al. 1994). Bioactive coatings such as hydroxyapatite enhance the biological response to not only metal implants (Ti6Al4V; Merolli et al. 2000) but also polymer implants (polyether ether ketone; Johansson et al. 2016), giving rise to tightly interlocked lamellar bone, with osteocytes in close apposition to the coating. Also supporting the formation of osteocyte-containing mineralized tissue are thin (≈10 nm) graphene oxide coatings on cp-Ti (Jung et al. 2016); multilayer coatings consisting of chitosan and hydroxyapatite, with the capacity for controlled release of bone morphogenetic protein 2 (Shah et al. 2013); degradable particulates such as synthetically produced α-tricalcium phosphate/octacalcium phosphate (Elgali et al. 2014); deproteinized bovine bone (Elgali et al. 2014); and autogenous bone fragments generated during implant site preparation (Shah and Palmquist 2017). This may be an important parameter to conclusively demonstrate whether newly formed tissue adjacent to a given biomaterial is bone or calcified cartilage—although the 2 processes are not mutually exclusive, since chondrocytes may contribute to the overall osteocyte pool (Hinton et al. 2017).

Preparation of the implant site with drilling tools creates a zone of apoptotic osteocytes around the osteotomy (Chen et al. 2017), the extent of which correlates directly with the drilling speed and the resulting thermal injury (Wang et al. 2017). Evidence suggests that, being mechanosensors, osteocyte-like MLO-Y4 cells induce osteoclastic differentiation under supraphysiologic loading (Fahlgren et al. 2018). The argument, therefore, is that at intermediate and/or long healing periods, osteocytes play an active role in the continued maintenance of osseointegration of (dental) implants. Here, we review the current knowledge regarding the adaptations of the osteocyte lacuno-canalicular network in the vicinity of implant biomaterials (Table).

**Table.** Summary of the Published Literature.

Material	Geometry	Surface Finish	Species	Site	Healing Period	Analytic Technique	Reference
cp-Ti	Screw shaped	M	Human	Tibia	7 to 20 mo	TEM	Serre et al. 1994
cp-Ti	Screw shaped	M + GB	Rabbit, NZW	Tibia	8 wk	Histology, CLSM	Piattelli et al. 1994
HAp	Coating on Ti6Al4V		Rabbit	Femur, MC	4 to 34 wk	BSE-SEM	Merolli et al. 2000
HAp	Coating on PEEK		Rabbit, SLE	Femur	3 to 12 wk	Histology	Johansson et al. 2016
Graphene oxide	Coating on cp-Ti		Rat, SD	Calvarium	8 wk	Histology	Jung et al. 2016
Chitosan + HAp	Coating on cp-Ti		Rat, SD	Tibia	1 to 4 wk	Histology	Shah et al. 2013
α-TCP + OCP; DBB	Particles		Rat, SD	Femur	3 to 28 d	Histology, BSE-SEM	Elgali et al. 2014
Autogenous bone	Particles		Rat, SD	Tibia	6 d	BSE-SEM	Shah and Palmquist 2017
cp-Ti	Screw shaped	M, M + dual AE	Rat, SD	Tibia	4 wk	Histology, RCE	Shah, Stenlund, et al. 2016
cp-Ti	Screw shaped	M, M + GB	Rat, SD	Maxilla	8 wk	RCE	Du, Ivanovski, et al. 2016
cp-Ti	Screw shaped	M, M + GB	Rat, SD	Maxilla	3 to 28 d	RCE, RT-qPCR	Du, Xiao, et al. 2016
cp-Ti	Screw shaped	M, M + laser ablation	Rabbit, NZW	Tibia	8 wk	RCE, HAADF-STEM	Shah, Johansson, et al. 2016
cp-Ti	Screw shaped	M + laser ablation	Human	Maxilla	4 y	Histology	Shah et al. 2014
cp-Ti	Screw shaped	M + laser ablation	Human	Maxilla	4 y	RCE, HAADF-STEM	Shah et al. 2015
Ti6Al4V; CoCr	Macroporous	EBM	Sheep	Femur	6 mo	BSE-SEM	Shah, Omar, et al. 2016
Ti6Al4V	Macroporous; cylinder	EBM, EBM + M	Sheep	Femur	6 mo	RCE	Shah, Snis, et al. 2016
45S5.6Sr glass	Particles		Rat, Wistar	Male	30 d	RCE	Gorustovich 2010
CoCr ± Zr	Cylinder	EBM	Rabbit, NZW	Femur	8 wk	BSE-SEM, RCE	Shah et al. 2018
TI6Al4V	Screw shaped	M	Mouse, WT	Femur	1 to 7 d	IHM	Cha et al. 2015
Ti6Al4V	Screw shaped	M	Rabbit, JW	Tibia	20^a^ wk	RCE	Sasaki et al. 2015
Ti6Al4V	Screw shaped	M	Rabbit, JW	Tibia	20^a^ wk	Histology	Kuroshima et al. 2015
Ti6Al4V	Screw shaped	M + ±60° grooves	Rabbit, JW	Tibia	20^a^ wk	Histology	Kuroshima et al. 2017
cp-Ti	Screw shaped	M	Rat, Wistar	Maxilla	8^b^ wk	Histology, IHM	Uto et al. 2017
cp-Ti	Screw shaped	GB + AE	Human	Alveolar bone, n.s.	4 wk to 27 y	Histology	Piattelli et al. 2014
cp-Ti	Screw shaped	GB + AE.	Human	Mandible	8 wk	Histology	Barros et al. 2009
cp-Ti	Cylinder	M	Rat, Wistar	Maxilla	5 d to 12 mo	Histology, IHM	Haga et al. 2011
cp-Ti	Cylinder	Dual AE	Rat, SD	Femur MC	2 to 8 wk	Micro-CT, pullout tests	Virdi et al. 2012
cp-Ti	Cylinder	Dual AE	Rat, SD	Femur MC	4 to 12 wk	Micro-CT, pullout tests	Virdi et al. 2015
cp-Ti	Cylinder	Dual AE	Rat, SD	Femur MC	12 wk	Micro-CT, pullout tests	Liu et al. 2012
cp-Ti	Screw shaped	AO	Rat, SD	Tibia	1 to 28 d	RT-qPCR	Lennerås et al. 2015
cp-Ti	Screw shaped	n.s.	Rat, Wistar	Tibia	4 to 28^c^ d	Histology, RT-qPCR	Zhang et al. 2014

AE, acid etched; AO, anodically oxidized; BSE-SEM, backscattered electron scanning electron microscopy; CLSM, confocal laser scanning microscopy; CoCr, cobalt chromium; cp-Ti, commercially pure titanium; DBB, deproteinized bovine bone; EBM, electron beam melting; GB, grit blasted; HAADF-STEM, high-angle annular dark-field scanning transmission electron microscopy; HAp, hydroxyapatite; IHM, immunohistomorphometry; JW, Japanese white; M, machined; MC, medullary canal; micro-CT, micro–computed tomography; n.s., not specified; NZW, New Zealand white; OCP, octacalcium phosphate; PEEK, polyether ether ketone; RCE, resin cast etching; RT-qPCR, reverse transcription quantitative polymerase chain reaction; SD, Sprague Dawley; SLE, Swedish lop-eared; TEM, transmission electron microscopy; TCP, tricalcium phosphate; WT, wild type; Zr, zirconium.

a Includes 12 wk of submerged healing prior to 8 wk of loading (50 N, 3 Hz, 1,800 cycles, 2 d/wk).

b Includes 3 wk of submerged healing prior to 5 wk of loading (10 N, 3 Hz, 1,800 cycles, 2 d/wk).

c Immediate (within 24 h) or delayed (after 28 d of submerged healing) loading.

### Direct Attachment to the Implant Surface

**Figure 2.**
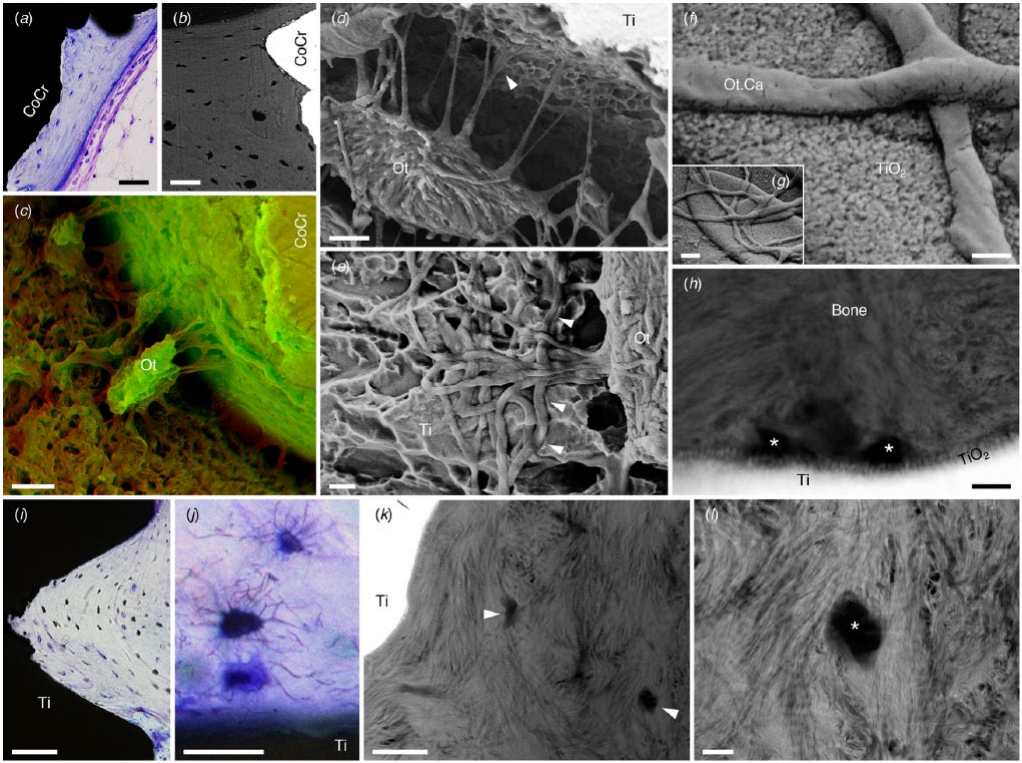
The proximity of osteocytes (Ot) to implant surfaces can be observed with (**a**) histology and (**b**) backscattered electron scanning electron microscopy. Scale bars: 50 µm. CoCr, cobalt chromium. (**c**) Techniques such as resin cast etching allow visualization of osteocyte attachment to implant surfaces. Scale bar: 5 µm. Here, scanning electron microscopy images obtained in the secondary electron mode at 5 kV (red channel) and 20 kV (green channel) have been merged to achieve contrast. (Adapted with permission from Shah, Omar, et al. 2016. Copyright 2016, Elsevier.) (**d**) Osteocyte attachment to an acid-etched micrometer-smooth surface. Scale bar: 2 µm. (**e**) Interdigitation of canaliculi with the implant surface (arrowheads). Scale bar: 1 µm. (Reproduced from Shah, Stenlund, et al. 2016 under the terms of the Creative Commons Attribution License.) (**f**, **g**) Canaliculi (Ot.Ca) adherent to the surface of a laser-ablated implant. Scale bars: 200 nm (f), 1 µm (g). (**h**) Cross-sectional view of canaliculi (asterisks) in close association with the thickened-surface TiO_2_ layer of a laser-ablated implant (high-angle annular dark-field scanning transmission electron microscopy). Scale bar: 200 nm. (Reproduced from Shah, Johansson, et al. 2016 under the terms of the Creative Commons Attribution License.) (**i**) Osteocytes align parallel to the lamellar direction, which closely follows the microcontour of the laser-ablated implant surface. Scale bar: 100 µm. (**j**) Adjacent to the implant surface, osteocytes form an interconnected network. Scale bar: 25 µm. (**k**) Canaliculi (arrowheads) can be identified within the first several micrometers from the implant surface (high-angle annular dark-field scanning transmission electron microscopy). Scale bar: 1 µm. (**l**) Mineralized collagen fibrils appear to “wrap around” the canaliculus (asterisk). Scale bar: 200 nm. (Adapted with permission from Shah et al. 2014. Copyright 2014, Elsevier.)

More recent evidence of osteocyte attachment has been provided with resin cast etching (Fig. 2). However, certain artifacts (e.g., tissue shrinkage attributable to sample preparation routines) induce the development of a narrow space between the implant surface and bone, which is subsequently filled by a thin film of the embedding medium, thus obscuring the direct osteocyte-implant contact (Shah, Stenlund, et al. 2016). Such artifacts are more frequent in relation to relatively smooth machined surfaces that afford little mechanical interlocking. With increasing complexity of the implant surface, osteocytes adjacent to topologically modified but micrometer-smooth (S_a_ = 456 nm) implants tend to become aligned with their long axes parallel to the implant surface, extending up to 20-µm-long canaliculi toward it (Shah, Stenlund, et al. 2016). Adjacent to microrough implants (R_a_ = 1.55 µm), osteocytes form an interconnected lacuno-canalicular system and attach to the implant surface through canaliculi (Du, Ivanovski, et al. 2016). Direct attachment of osteocytes to minimally rough (S_a_ = 519 nm) and microrough (S_a_ = 906 nm) implants has also been demonstrated in ovariectomized and sham-operated conditions (Du, Xiao, et al. 2016). Where the dimensions of implant surface features are appropriate (e.g., submicron topography achieved through acid etching), canaliculi may become closely interdigitated with the topographic features (Shah, Stenlund, et al. 2016).

In the case of laser-ablated implants, where a thick-surface TiO_2_ layer (~50 nm) is superimposed over globules (2 to 10 µm) of resolidified metal, canaliculi extend several micrometers, branch, and remain attached to the implant surface in spite of removal torque measurements (generally performed in a preclinical model; Shah, Johansson, et al. 2016). Adjacent to functionally loaded, laser-ablated dental implants in human, osteocytes closest to the implant surface are aligned parallel to both the lamellar direction and the microscale contour of the implant surface (Shah et al. 2014). Their associated canaliculi, directed perpendicularly, extend toward the implant surface and branch in close proximity to the ~200-nm-thick channel-like surface TiO_2_ layer and extend for tens of micrometers toward Haversian canals (Shah et al. 2015).

**Figure 3.**
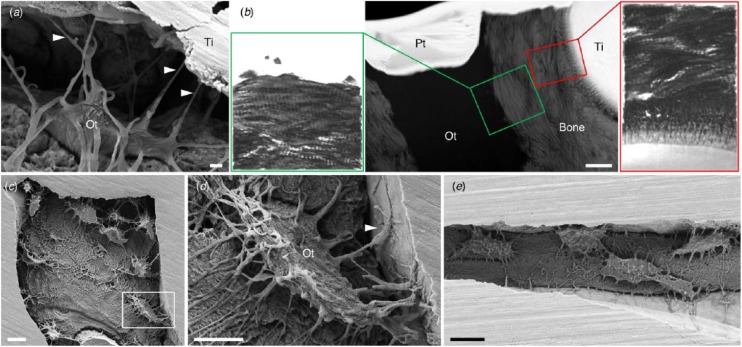
Direct attachment of osteocytes to the implant surface. (**a**) Osteocytes (Ot) retain connectivity to the implant surface after 4 y of clinical function through canaliculi (arrowheads). (**b**) Ultrastructural similarities exist between the bone-implant interface and the bone-osteocyte interface (high-angle annular dark-field scanning transmission electron microscopy and electron tomography), where both comprise highly aligned collagen fibrils forming typical rope-like bundles. Scale bar: 1 µm. (Adapted with permission from Shah et al. 2015. Copyright 2015, American Chemical Society.) (**c**) Interconnected osteocyte lacuno-canalicular network within 60-µm-wide features on the surface of 3D printed Ti6Al4V. (**d**) An osteocyte (box in c) attaches to the implant surface through numerous branching canaliculi (one of which is indicated by an arrowhead). (**e**) Interconnected osteocytes within a 14-µm-wide crevice on the surface of 3D printed Ti6Al4V. (Adapted with permission from Shah, Snis, et al. 2016. Copyright 2016, Elsevier.) Scale bars: 10 µm (c, e), 5 µm (d). Pt, platinum; Ti, titanium.

Interconnectivity among neighboring osteocytes and formation of extensive lacuno-canalicular networks in the vicinity of the implant surface indicate cell-to-cell communication (Fig. 3). Interestingly, osteocytes located within a few micrometers of the implant surface show an arrangement of mineralized collagen fibrils at the bone-osteocyte interface similar to that observed at the bone-implant interface (Shah et al. 2015). Osteocyte attachment to highly complex, 3-dimensionally (3D) printed macroporous geometries has also been reported (Shah, Omar, et al. 2016; Shah, Snis, et al. 2016).

Besides sample preparation artifacts, removal torque measurements for determining biomechanical anchorage of implants may preclude direct visualization of osteocyte attachment to the implant surface (Shah, Johansson, et al. 2016). Degradable materials such as bioactive glass (Gorustovich 2010), where the bone-implant interface tends to migrate, support osteocyte attachment via dendritic processes that appear to pass through the Ca-P-rich interfacial layer and reach the silica-rich surface of the reacted bioactive glass.

Apropos of the extensiveness of the lacuno-canalicular network, its attachment and close adaptation to the implant surface may serve as a physical factor contributing toward strong bone-implant interlocking. Furthermore, a dense, well-aligned network of dendritic processes in the vicinity of the implant surface may be able to detect compressive and tensile strains imposed on the interfacial tissue, allowing for structural adaptations to maintain homeostasis.

### Osteocyte Survival and Density

Within newly formed tissue adjacent to microrough implants (Du, Ivanovski, et al. 2016) and 3D printed solid and macroporous Ti6Al4V implants (Shah, Snis, et al. 2016), the osteocyte density (N.Ot/B.Ar) is higher than in native lamellar bone distant from the implant. Not surprising, newly formed peri-implant tissue is compositionally less mature than native lamellar bone (Shah, Snis, et al. 2016) and therefore may contain yet unremodeled areas of woven bone contributing to the higher osteocyte densities. However, N.Ot/B.Ar values in the newly formed peri-implant bone may be less sensitive to changes in material composition—such as macroporous Ti6Al4V versus CoCr (cobalt chromium) implants (Shah, Omar, et al. 2016) and cylindrical CoCr ± zirconium implants (Shah et al. 2018)—or surface topography, including machined versus laser-ablated implants (Shah, Johansson, et al. 2016).

Around the osteotomy site, a zone of dead and/or dying osteocytes can be observed through staining methods such as DAPI (4′,6-diamidino-2-phenylindole) to detect cell nuclei and TUNEL (terminal deoxynucleotidyl transferase dUTP nick end labeling) to detect apoptotic cells (Cha et al. 2015). Increasing the zone of dead and/or dying osteocytes results in more extensive bone resorption and slower bone formation (Cha et al. 2015). In addition to other factors, insertion torque is affected by the diameter of an implant relative to the osteotomy site. In murine femora, it is estimated that low insertion torque (0.05 ± 0.03 N·cm) induces a ~75- to 90-µm-wide zone of cell death, while high insertion torque (0.18 ± 0.02 N·cm) induces a much wider zone of cell death, ~145 to 165 µm, as a result of lateral compression by the implant (Cha et al. 2015).

**Figure 4.**
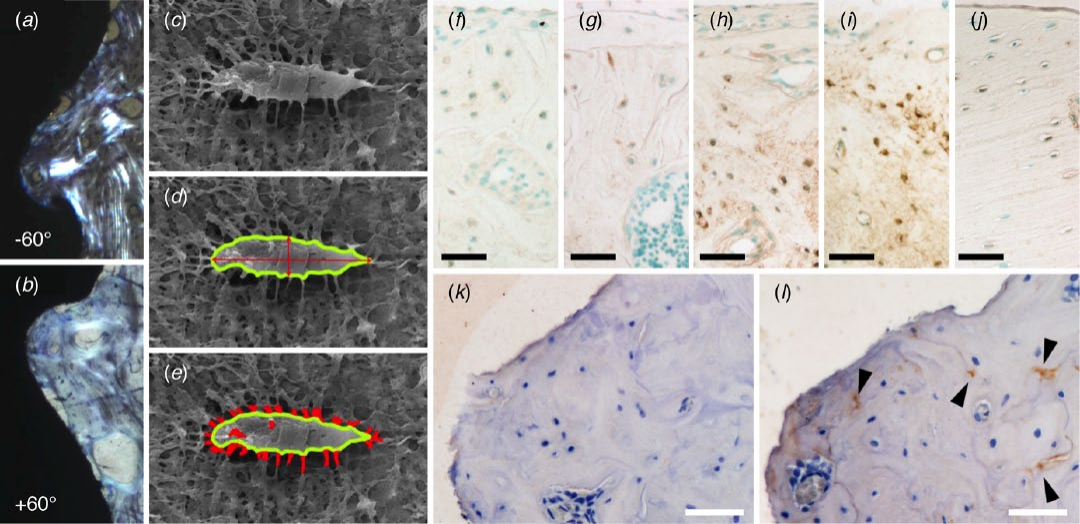
Mechanical loading influences collagen alignment, osteocyte morphology, and the expression and production of osteocyte-related genes and proteins. (**a**, **b**) Polarized light microscopy reveals a preferential alignment of collagen fibers. The angle difference between groove direction and alignment direction of collagen fibers in +60° groove is smaller than in −60° groove. The grooves are 400 μm in pitch and 200 μm in depth. (Adapted with permission from Kuroshima et al. 2017. Copyright 2017, Elsevier.) (**c–e**) Assessment of osteocyte morphology via resin cast etching: (d) aspect ratio and (e) dendricity. (Adapted with permission from Sasaki et al. 2015. Copyright 2017, Elsevier.) Sclerostin immunoreactivity in peri-implant bone at (**f**) 5 d, (**g**) 10 d, (**h**) 20 d, (**i**) 2 mo, and (**j**) 7 mo. Scale bars: 35 µm. (Adapted with permission from Haga et al. 2011. Copyright 2011, John Wiley and Sons.) Influence of cyclic mechanical loading on the production of the osteoprotective gene *Sema3A*; as compared with (**k**) unloaded conditions, loading can increase (**l**) Sema3A production (arrowheads). Scale bars: 50 µm. (Reproduced from Uto et al. 2017 under the terms of the Creative Commons Attribution License.)

In mechanically loaded conditions (Fig. 4), cyclic loading has been shown to increase not only the bone volume up to 500 µm around osseointegrated implants but also the N.Ot/B.Ar (Kuroshima et al. 2015; Sasaki et al. 2015), and it affects the alignment of collagen and the crystallographic *c*-axis of bone apatite (Kuroshima et al. 2017). At the upper part of the implant (cortical bone level), N.Ot/B.Ar was ~55% higher under loading, as measured with 10-µm-thick histologic sections (Kuroshima et al. 2015). A similar pattern has been reported for measurements made with resin cast etching. At the upper part of the implant (cortical bone level), N.Ot/B.Ar increased by ~43% under loading (Sasaki et al. 2015). N.Ot/B.Ar may also vary depending on the vertical level along the implant where measurements are made, presumably due to differences in stress concentration patterns. Compared with measurements at the implant neck, N.Ot/B.Ar at the lower part of the implant (marrow cavity level) increased by ~71% under loading (Sasaki et al. 2015).

Depending on whether implant threads are located in cortical bone or trabecular bone, interthread variation in the number of osteoblasts per bone surface (N.Ob/B.S) and N.Ot/B.Ar has been reported in response to cyclic mechanical loading (Uto et al. 2017). Certain design features may alter the transmission patterns of externally applied loads. For instance, grooves (400 µm and 200 µm in pitch and depth, respectively) oriented at +60° (downward) or –60° (upward) to a plane perpendicular to the long axis of Ti6Al4V implants have been shown to influence the preferential alignment of collagen and the crystallographic *c*-axis of bone apatite (Kuroshima et al. 2017). Under loading, although bone volume increases irrespective of groove orientation, the effect on N.Ot/B.Ar is inequivalent, where N.Ot/B.Ar increase is noted for +60° grooves but not for –60° grooves, suggesting that certain specific optimal implant design features could control bone quality (as interpreted from osteocyte densities and the alignment of the building blocks of bone; Kuroshima et al. 2017).

Around clinical dental implants retrieved after different loading periods, one study reported variation in N.Ot/B.Ar, ranging from 415 ± 129 mm^-2^ at 1 to 7 mo, increasing to 1,620 ± 282 mm^-2^ at 1 to 5 y, and finally declining to 426 ± 129 mm^-2^ at 14 to 27 y (Piattelli et al. 2014). The explanation given for the decrease in N.Ot/B.Ar observed after extended in vivo durations is that once bone structure is well aligned and biomechanically competent, fewer osteocytes may be necessary to maintain tissue homeostasis under loading (Piattelli et al. 2014). Also attributed to functional adaptation in response to the loading stimulus, peri-implant bone around immediately loaded implants tends to contain a higher N.Ot/B.Ar than around implants that have undergone submerged healing prior to loading (Barros et al. 2009).

### Osteocyte Morphology and Dendricity

In woven bone, formed rapidly after implantation, round-shaped osteocytes are embedded irregularly; however, a well-arranged organization of the osteocyte lacuno-canalicular system is gradually attained through remodeling (Haga et al. 2011).

Repetitive mechanical loading has been shown to influence cell morphology, resulting in an increased average number of canaliculi per osteocyte lacuna (N.Ot.Ca/Ot.Lc) and decreased osteocyte ellipticity (Sasaki et al. 2015). However, in unloaded experimental setups, a disorganized arrangement of osteocytes has been noted in areas of new bone adjacent to microrough implants, as compared with areas of native lamellar bone (Du, Ivanovski, et al. 2016) . In ovariectomized and sham-operated rats, osteocytes adjacent to minimally rough implants and microrough implants also exhibited a disorganized arrangement and appeared less elliptical than those in areas of native lamellar bone (Du, Xiao, et al. 2016).

After long-term submerged healing in sheep, the N.Ot.Ca/Ot.Lc adjacent to the surface of 3D printed, macroporous, and solid Ti6Al4V implants was 38% to 42% higher than in areas of native lamellar bone (Shah, Snis, et al. 2016). Here also, osteocytes adjacent to the implant surface were less elliptical but slightly larger than those in areas of native lamellar bone (Shah, Snis, et al. 2016).

In bone adjacent to functionally loaded, laser-ablated clinical dental implants, the N.Ot.Ca/Ot.Lc close to the implant surface (25 ± 6 mm^-2^) is reportedly higher than in areas of older bone external to the implant thread (17 ± 3 mm^-2^), indicating less aged tissue adjacent to the implant surface (Shah et al. 2015).

### Expression and Production of Osteocyte-Related Genes and Proteins

Studies have investigated the expression of osteocyte-related genes. For instance, *DMP1* expression in response to microrough implants is upregulated relative to minimally rough implants at very early healing (i.e., 3 d in ovariectomized and sham-operated conditions). At 7 d, *DMP1* expression in sham-operated conditions is upregulated relative to ovariectomized conditions in response to microrough implants only (Du, Xiao, et al. 2016). *SOST* expression up to 7 d, however, remains unaffected by minor variations in implant surface roughness (Du, Xiao, et al. 2016).

Reportedly, sclerostin production can be correlated to the relative age of bone formed around metal implants. In newly formed woven bone, a small number of osteocytes stain positive for sclerostin, thus indicating immature tissue. At intermediate healing times, sclerostin staining becomes more intense, implying an inhibitory influence on osteoblastic activity. Eventually, once a steady state is attained, sclerostin staining is minimal, suggesting that there is no further need to modulate osteoblastic activity (Haga et al. 2011).

Blockade of the protein sclerostin through subcutaneous administration of sclerostin antibodies may, however, improve biomechanical anchorage of cylindrical implants (Virdi et al. 2012). This approach is also particularly effective in ovariectomized and sham-operated conditions (Virdi et al. 2015). Moreover, sclerostin blockade is effective in preventing particle-induced implant loosening (Liu et al. 2012).

As compared with machined surfaces, the physicochemical properties of anodically oxidized surfaces stimulate bone turnover through regulation of RANKL, RANK, and OPG and, therefore, the RANKL/OPG expression ratio of implant-adherent cells (Lennerås et al. 2015). The RANKL/OPG ratio appears to be influenced by mechanical loading. Application of cyclic mechanical loading to micrometer smooth (R_a_ = 0.45 µm) implants after a period of initial submerged healing has been shown to decrease the RANKL/OPG ratio, suggesting a stimulatory effect of dynamic loading on implant osseointegration and molecular adaptations that favor bone formation and simultaneously affect bone remodeling (Zhang et al. 2014).

Externally applied cyclic loading can increase production of the osteoprotective gene *Sema3A*, with corresponding increases in N.Ob/B.S, N.Ot/B.Ar, and collagen area fraction, in addition to a preferential alignment of collagen fibers (Uto et al. 2017). These effects, in the vicinity of the implant surface, have been attributed to distinct stress distribution patterns.

## Current Concepts

**Figure 5.**
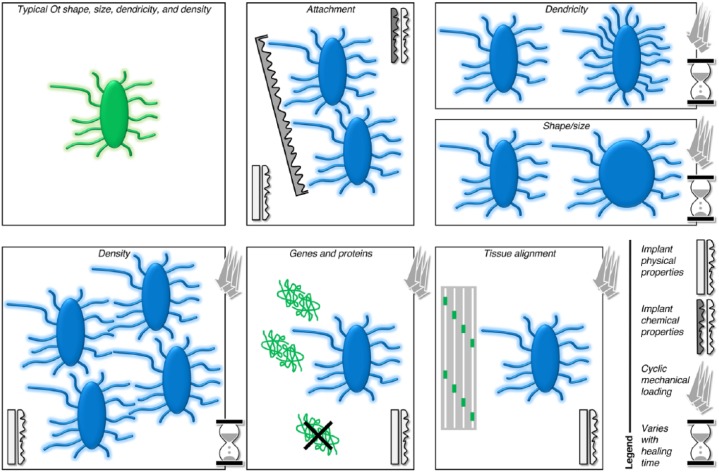
The impact of various factors—including implant physical and chemical properties (e.g., surface topography, bulk material), mechanical loading conditions, and healing time—on the morphology and resulting function of the osteocyte lacuno-canalicular system in the vicinity of implant biomaterials.

In the vicinity of implant biomaterials, osteocytes respond to local environmental factors in a variety of ways (Fig. 5). Similar to normal bone, the osteocyte lacuno-canalicular network in the vicinity of osseointegrated implants represents the bone formation patterns and, in particular, the origins of lamellar structure of bone adjacent to the implant surface (Shah, Stenlund, et al. 2016). Certain morphologic parameters of osteocytes (e.g., size, alignment with respect to bone lamellae, density, proximity to small and large blood vessels, lacuno-canalicular interconnectivity between neighboring and distant osteocytes) disclose vital clues regarding the status of bone, as tissue regeneration occurs in the vicinity of, and in response to, implanted biomaterials (Shah et al. 2014; Shah, Snis, et al. 2016; Shah, Stenlund, et al. 2016).

An increased number of osteoblasts and, consequently, higher osteocyte density in response to mechanical loading indicate that not only recruitment but also survival of cells may be externally controlled (Uto et al. 2017). This effect is particularly exaggerated where implant design features include grooves directed +60° (downward) to a plane perpendicular to the long axis of the implant (Kuroshima et al. 2017), which is considered optimal for achieving an anisotropic principal stress distribution and preferential alignment of collagen and the crystallographic *c*-axis of bone apatite, parallel to the groove direction (Noyama et al. 2013). However, osteocyte apoptosis induced by mechanical forces results in extensive bone remodeling and minimal new bone formation (Cha et al. 2015).

There is an apparent discrepancy among osteocyte densities reported by different studies, which is attributable to factors such as loading conditions (e.g., frequency, cycles, types, and magnitude), interspecies variation, healing durations, and analytic methods. Generally, resin cast etching reveals more osteocytes than backscattered electron scanning electron microscopy, while measurements made with optical microscopy are influenced by the thickness of histologic sections. In terms of organization and alignment of the building blocks of bone, ultrastructural similarities between the bone-osteocyte interface and the bone-implant interface indicate that certain metals and alloys tend to elicit bone bonding and remodeling similar to normal bone formation (Shah et al. 2015).

## Future Perspectives

In addition to being indicators of bone quality, osteocytes are important structural markers of osseointegration and can prove exceptionally valuable in characterizing the biological response to currently available and novel materials for load-bearing applications in dental rehabilitation and orthopedics. Despite a clinical success rate >95%, knowing the status of the host bed prior to implant placement may be advantageous in certain situations. In compromised conditions, for instance, could radiologic assessment be accompanied by another parameter? Recent developments in third harmonic generation microscopy have made label-free (Genthial et al. 2017), intravital (Tokarz et al. 2017) imaging of the osteocyte lacuno-canalicular network possible. We therefore propose that evaluation of peri-implant osteocytes should be a necessary component in future investigations of osseointegration. Particular emphasis must be placed on investigating the physical and chemical interactions among osteocytes and more recently developed bioceramics, polymers, and composites. Specifically, questions to address include the following: 1) How do osteocytes respond to stimuli other than mechanical loading (e.g., bacteria) on the molecular level? 2) Could sudden high-impact trauma lead to a cascade of structural deterioration and implant loosening? 3) What is the role of osteocytes in bone maintenance around biomedical implants in disease states? 4) Is peri-implantitis associated with changes in the osteocyte lacuno-canalicular network?

## Author Contributions

F.A. Shah, contributed to conception, design, and data interpretation, drafted and critically revised the manuscript; P. Thomsen, A. Palmquist, contributed to conception, design, and data interpretation, critically revised the manuscript. All authors gave final approval and agree to be accountable for all aspects of the work.
